# Antibiotics release by hybrid bone scaffold: relationship between kinetic profiles and scaffold intrinsic features 

**DOI:** 10.3389/fbioe.2025.1679920

**Published:** 2025-12-18

**Authors:** Marta Tavoni, Federico Pupilli, Elisa Restivo, Laura Grillini, Elisabetta Galassi, Anna Tampieri, Simone Sprio

**Affiliations:** 1 Institute of Science, Technology and Sustainability for Ceramics, National Research Council of Italy (ISSMC-CNR), Faenza, Italy; 2 Molecular Medicine Department (DMM), Centre for Health Technologies (CHT), Unità di Ricerca (UdR) INSTM, Operative Unit (OU) of the Interuniversity Center for the Promotion of the 3Rs Principles in Teaching and Research (Centro 3R), University of Pavia, Pavia, Italy; 3 Finceramica Faenza SpA, Faenza, Italy

**Keywords:** antibacterial effect, antibiotics, bone regeneration, bone scaffold, collagen, hydroxyapatite, kinetic release, *Staphylococcus aureus*

## Abstract

Bone infections are a major complication in the treatment of bone defects, often leading to chronic conditions such as osteomyelitis and prosthetic joint infections, predominantly caused by *Staphylococcus aureus* bacteria. Whilst antibiotics are essential to infection control, systemic administration often fails to achieve effective concentrations at the infection site, increasing the risk of toxicity and antimicrobial resistance. In this study we propose a hybrid, scaffold obtained by a bio-inspired mineralization process (HS), designed to support bone healing and enabling localized antibiotic delivery. The HS consist of nanocrystalline magnesium-doped apatite nanocrystals heterogeneously nucleated on self-assembling collagen fibrils, mimicking natural bone mineralization processes. The scaffold is subsequently tested for its ability to modulate the release of vancomycin, gentamicin, and tobramycin and evaluate their efficacy in inhibiting *Staphylococcus aureus* growth by agar diffusion test. Antibiotic loading using clinically applicable methods and tracking their release over time was inspected and the experimental data was analysed using pseudo-first and pseudo-second order kinetics, showing pathways related to HS chemistry, structure, and drug physicochemical properties. Compared to burst antibiotic releases observed in sintered apatite scaffold, the hybrid scaffold demonstrated a more controlled and sustained release of antibiotics. Our findings highlight how scaffold nanostructure and surface characteristics can influence drug release, with regenerative capacity and sustained local antibiotic delivery potentially improving bone repair by reducing post-surgical infections.

## Introduction

In the surgical treatment of bone defects affected by trauma or degenerative diseases such as tumours the occurrence of postoperative infections represents a significant complicating factor, potentially leading to therapy failure, encompassing osteomyelitis (OM), prosthetic joint infections (Cazalbou et al., 2015; Li and Webster, 2018; Shuaishuai et al., 2023) and bone resorption (Vallet-Regí and Arcos, 2013; Masters et al., 2022). The current treatment of bone infections involves the systemic administration of antibiotics for long periods or, in the worst cases, the surgical removal of infected/necrotic bone (Alegrete et al., 2023). However, in several cases the formation of bacterial biofilms provides a physical and impermeable barrier to immune cells and therapeutic agents, preventing the antibiotics effectiveness. As a result, the dosage of antibiotics required to treat biofilm-forming bacteria is up to 1,000 times higher than for planktonic one (Vallet-Regí and Arcos, 2013). Despite being the most important and effective methods of treating bone infections, systemic antibiotics administration cannot target the surgical site, and determining the appropriate dosage still remains a critical issue (Cazalbou et al., 2015; Shuaishuai et al., 2023). Besides adverse reactions, high doses of antibiotics can lead to antimicrobial resistance, which is one of the most global public health and economic threat that may be accelerated by the overuse of antibiotics worldwide (Ahmad and Khan, 2019; Hutchings et al., 2019; Sprio et al., 2019). In this regard, vancomycin (VNC), a glycopeptide antibiotic able to inhibit bacterial cell-wall synthesis, is one of the most used antibiotic in clinics for treating serious infections caused by gram-positive bacteria, including methicillin-resistant *Staphylococcus aureus* (MRSA) (Cheung and DiPiro, 1986; Rybak et al., 2009; Bruniera et al., 2015). However its systemic administration requires careful attention due to its potential nephrotoxicity and other side effects (Filippone et al., 2017; Jeffres, 2017). Other broad-spectrum antibiotics frequently employed in the clinical practice include gentamicin (GNT) and tobramycin (TBR) which can inhibit bacterial protein synthesis. Both GNT and TBR are aminoglycoside antibiotics with overlapping but distinct antibacterial spectra, and both can cause ototoxicity and nephrotoxicity (Kumin, 1980).

In this view, the development of new solutions for tissue regeneration coupled with drug delivery ability is today a very active area of research (Ratier et al., 2004; Ke Ren, 2014; Grigore, 2018; Chindamo et al., 2020; Liao et al., 2021; Dapporto et al., 2022). Nowadays, it is widely accepted that the regeneration of critical bone defects requires the use of scaffolds acting as an instructive guide for endogenous cells to promote bone regrowth and remodeling (Tampieri et al., 2016; Ruffini et al., 2021; Tavoni et al., 2021; Pupilli et al., 2022). In this regard, key aspects for bone scaffolds are their compositional and structural mimicry with host tissues, relevant to prevent adverse reactions and promote cell adhesion/proliferation, osteogenic differentiation and the formation of new bone tissue firmly integrated with the scaffold (Yang et al., 2011; Xiao et al., 2020). This implies that the scaffold should exhibit appropriate physico-chemical and structural properties (Hu et al., 2007; Yang et al., 2011; Costa et al., 2013; Zan et al., 2016; Sprio et al., 2019; Maji and Mondal, 2020; Xiao et al., 2020; Zhu et al., 2020; Dapporto et al., 2022), able at the same time to promote drug binding and controlled *in situ* release in order to ensure efficient on-site therapies (Lanzillotti et al., 2024).

Several previous studies demonstrated that nanocrystalline and ion-substituted apatites display remarkable osteogenic, osteointegrative and bioresorption capabilities, largely due to their compositional similarity to the mineral phase of bone (Wu et al., 2014; Sprio et al., 2016; Ballardini et al., 2017; Mazzoni et al., 2020; Iaquinta et al., 2021; Kon et al., 2021). Ion-doped nano-apatites exhibit high specific surface areas and electrically charged groups at the surface that facilitate strong interactions with foreign molecules, including drugs, growth factors and nucleic acids (Chen et al., 2020; Sun et al., 2020; Mulazzi et al., 2021; Dapporto et al., 2022; Lanzillotti et al., 2024). However, a significant constraint in the development of nanocrystalline apatite scaffolds is associated with the necessity of thermal treatments for effective material consolidation. Such a procedure yields the growth of apatite crystals, leading to the segregation of doping ions into secondary phases, thus resulting in a considerable reduction in the specific surface area, bioactivity, bioresorbability and, ultimately, the regenerative ability (De Carvalho et al., 2019). For this reason, various studies in the past years were dedicated to the development of new sinter-free processes able to generate 3D biomimetic bone scaffolds. In this respect, 3D printed ceramic/polymer composite scaffolds can be obtained with nanocrystalline apatite powders. However, the non-metabolic dissolution of bio-erodible (non-biomimetic) polymers composing the scaffold poses various concerns on osteointegrability and quality of newly formed bone (Liu et al., 2006). Relevant examples of biomimetic scaffolds prepared at low temperature are given by apatitic bone cements, obtained by dissolution/reprecipitation processes acting at body temperature, endowed with high osteointegrative ability and also able to link and release antibiotic and antitumor drugs (Sprio et al., 2016; Dapporto et al., 2022; Pylostomou et al., 2023). Alternative methodologies have explored low temperature aqueous processes carried out at body temperature to obtain the heterogeneous nucleation of nano-apatites directly on Type I collagen. This phenomenon was observed to occur alongside the self-assembling of collagen molecules into a highly interconnected nano-fibrillary structure, as driven by pH variation, with the aim of mimicking the natural biomineralization process leading to bone formation in mammals (Sprio et al., 2014; Krishnakumar et al., 2018). This process yielded highly porous 3D bio-hybrid scaffolds with soft and malleable structure, where the content of the mineral phase could be tailored, during the synthesis process, to reach bone-like levels (Tampieri et al., 2008; Sprio et al., 2014; Krishnakumar et al., 2017; Krishnakumar et al., 2018; Mulazzi et al., 2021). A relevant aspect is related to the highly disordered microenvironment offered by such a bio-hybrid scaffold, mimicking the newly formed bone composition and structure (Scaglione et al., 2012), where the nearly amorphous character and multiple ions doping characterizing the mineral phase are a source of high osteogenic properties and ability of metabolic bio-resorption. Indeed, such a scaffold was previously used in various clinical trials focused on bone regeneration in different anatomical districts such as the spine, the hip and the oral cavity for purpose of sinus augmentation or socket preservation, showing excellent osteointegrative and regenerative ability (Grigolo et al., 2016; Mozzati et al., 2017; Scarano et al., 2017; Taschieri et al., 2019; Barbanera et al., 2020; Gioia et al., 2020; Cimatti et al., 2022).

To face different clinical cases, this scaffold was soaked into antibiotics prior to implantation, for the treatment of cavitary or segmental bone defect in 13 patients affected by a broad spectrum of pathologies, including septic non-union, chronic osteomyelitis and peri-prosthetic joint infection (Romanò et al., 2015) (note: the device was used outside of its IFU and surgical guidelines, which require clearance of the implant site from infection prior to scaffold grafting). After 24 months no adverse events nor infections were detected and satisfactory bone healing was experienced by 10 patients, thus showing that the approach of intra-operative treatment with antibiotic-loaded scaffolds is feasible and promising, thus encouraging the development of defined and effective clinical protocols.

In this respect, the present work illustrates the controlled-release ability of hybrid scaffolds (HS) obtained by heterogeneous nucleation of apatite nanocrystals partially substituted with Mg^2+^ and CO_3_^2-^ ions on self-assembling Type I collagen fibrils derived from equine tendons. The HS was loaded with vancomycin hydrochloride (VNC), gentamicin (GNT) or tobramycin (TBR), via simple and clinically applicable procedures. The VNC release process was analysed with HS in comparison with a macroporous sintered apatite scaffold (MSS), in relation to their specific physical-chemical characteristics under physiological conditions. Then, the kinetic releases of VNC, GNT and TBR from the HS were analysed and compared to highlight the different chemical interactions between the scaffold and the antibiotics, as modulated by their different molecular structure and chemical reactivity, particularly to assess the ability of HS to release different types of antibiotics in a sustained manner. The efficacy of antibiotics’ release from HS scaffolds has been tested through agar diffusion test against *Staphylococcus aureus* which is one of the most common pathogens that causes bone infections (Masters et al., 2022).

Despite the growing interest in calcium phosphate-based scaffolds for biomedical applications, relatively few studies have specifically addressed the mechanisms governing drug release from these materials. A comprehensive understanding of the release kinetics and the underlying factors is essential for optimising the therapeutic efficacy of these devices, particularly in the context of antibiotic delivery aimed at preventing or treating infections associated with implantable devices. This work aims to highlight how key factors such as scaffold composition, porosity, and drug–matrix interactions are directly related to the release of antibiotics. Clarifying these mechanisms will support the rational design of scaffolds with controlled drug release, improving outcomes in bone regeneration and infection prevention.

## Experimental methods

### Production of the macroporous sintered scaffold

Macroporous sintered scaffold (MSS) was prepared as previously reported (Dapporto et al., 2016). Briefly, commercial hydroxyapatite (HA: Honeywell International, Charlotte, NC) powder was calcined at 1,000 °C for 4 h. The powder was then dispersed in water using Dolapix CA (Zschimmer & Schwarz Chemie GmbH, Lahnstein, Germany) at a weight ratio HA:H_2_O:dispersant = 73:23:4. The suspension was prepared in a 250 mL zirconia jar with zirconia balls (15 mm diameter) using a high-energy milling treatment (planetary ball mill). Subsequently, 1%–3% Ammonium Lauryl Sulphate (Merck KGaA, Darmstadt, DE) was added to the suspension as foaming agent. After 15 min of rapid stirring, the resulting foamed suspension was poured into paper moulds and completely dried at room temperature to obtain stable ceramic foams. Finally, the samples were sintered at 1,200 °C for 1 h and cut into parallelepipeds (20 × 35 × 5 mm in size).

### Production of the hybrid scaffold

The production of the hybrid scaffold (HS) based on Mg^2+^-doped HA/collagen (40:60 wt.%) was prepared in accordance with the procedure previously reported (Di Martino et al., 2025). Briefly, equine type I collagen (Opocrin Group SpA, Modena, Italy) at 1%wt concentration was combined with phosphoric acid aqueous solution (H_3_PO_4_ 85 wt.% pure, Carlo Erba Reagents srl, Cornarendo (MI), Italy), obtaining a homogeneous acid collagen suspension. The acid suspension was then dropped into a basic suspension containing calcium hydroxide (Ca(OH)_2_, 95% pure, Carlo Erba Reagents srl, Cornaredo Italy) and magnesium chloride (MgCl_2_ 6H_2_O, Merck KGaA, Darmstadt, Germany). The precipitated mineralized collagen fibers were matured and washed with highly purified water. In order to stabilize the scaffold structure, the biomineralized collagen was then chemically crosslinked by immersion in a 0,05 wt% 1,4-butanediol diglycidyl ether (BDDGE, Merck KGaA, Darmstadt, Germany) solution, and subsequently washed with purified water. The scaffold was freeze-dried with a controlled freezing and heating ramp from 25 °C to −35 °C and from −35 °C to 25 °C, over a period of 25 h under vacuum conditions (P = 0.29 mbar) and finally gamma-sterilized at 25 kGy. Scaffold size: 25 × 35 × 5 mm.

### Drugs loading

A preliminary experiment was conducted to assess the maximum amount of water that can be absorbed by the MSS and HS. The water adsorption capacity of these scaffolds was evaluated by measuring the change in weight of each device after immersing them in double-distilled water for different periods of time. The immersion process was continued until the weight change stabilized, indicating that the maximum water adsorption had been reached. Subsequently, 80% of the maximum water absorption capacity was utilized for loading a VNC solution with a concentration of 50 mg/mL onto the scaffold. This loading was performed by using a pipette, with 1.8 mL of VNC solution applied to the MSS and 2.3 mL to HS. The impregnation process was carefully carried out through dropwise application of the solution onto the scaffold surface to ensure uniform distribution of the drugs. At the same time, GNT and TBR were exclusively loaded onto the HS. In this instance, the drug loading protocol was the same of VNC using a 40 mg/mL GNT or TBR solution.

### Drugs release and detection

After the functionalization, the drug-loaded samples (weight: 0.9 g) were deposited inside 50 mL falcon tubes filled with 20 mL of PBS (phosphate saline 7.4 buffer). The release of antibiotics was monitored at various experimental times, by taking an aliquot (20%) of the eluate: 30 min, 1 h, 3 h, 6 h, 24 h and 48 h, renewing the buffer liquid with additions of 20% after each withdrawal. Prior to further analysis, the withdrawn solution was passed through a 0.22 μm syringe filter, to eliminate impurities. Experiments were carried out in triplicate for each drug tested and non-medicated scaffolds were incubated in duplicate to have a suitable reference.

The quantification of VNC released from the scaffolds was investigated by UV-Visible spectroscopy using a LAMBDA™ 750 UV/Vis/NIR spectrophotometer (PerkinElmer). A calibration curve absorbance vs. concentration was collected by measuring standard solutions obtained from the dilution of the mother solution, considering the molar extinction coefficient of VNC. The detection wavelength of VNC was 281 nm. Since GNT and TBR are not detectable in the UV region, further functionalization with a suitable chromophore has been necessary to observe their release kinetic. A modified spectrophotometric method inspired by previous works (Ismail et al., 2016; Klicova et al., 2023) has been applied in this study to determine the antibiotic quantitative release in the form of ninhydrin-drug complexes. Chemical interaction between ninhydrin and the tested drugs is based on the chemical interaction of ninhydrin with the primary and secondary amine groups present in the chemical structure of the observed drugs which produces purple colour (Manchón et al., 2015). The colorimetric reagent was prepared as follows: 50 mg of weighted ninhydrin powder was dissolved in 10 mL of phosphate buffered saline (PBS) solution (pH 7.4) to get a 5 mg/mL stock solution. The stock solution was prepared freshly before the analysis and kept under 4 °C, protected from any light source.

For the experiments, the quantification limits for GNT and TBR were determined and calibration curves with standard GNT and TBR stock solutions. The experimental procedure for the formation of GNT-ninhydrin complex (including blank samples and calibration solutions) proceeds as follows: equal amount of colorimetric reagent was added to the GNT solution before it was subjected to heat treatment in an oil bath (95 °C, 15 min) and followed with cold treatment using ice cold water bath for 5 min. TBR-ninhidrin spectrophotometric determination was achieved following the same experimental procedure, with a shorter heat treatment (95 °C, 10 min), as TBR was observed to be more reactive towards the formation of TBR-ninhydrin complex and degraded more rapidly than GNT. A blank sample of equal volumes of PBS and ninhydrin solution was prepared for UV spectrophotometry. After the derivatization procedure, quantitative analysis was achieved with a UV-Vis Spectrophotometer (NanoDropTM One/Onec Microvolume, Thermo Fisher Scientific, Waltham, MA, United States), selecting absorption maximum at 570 nm due to the colorimetric appearance of the solution (clear purple) and the obtained values were confronted with the calibration curve obtained with standard GNT and TBR solutions at known concentrations. The measurements were repeated five times for each sample and performed in triplicate for each drug.

The obtained data have been tentatively fitted to the kinetic models frequently encountered (Bhasarkar and Bal, 2019; Craciun et al., 2019; Zhang et al., 2019; Hong et al., 2024), namely the pseudo-first-order (Equation 1) and pseudo-second-order models (Equation 2):

$$F_{t}=1-e^{{-k}_{1}t}$$ (1)

$$F_{t}=\frac{k_{2}q_{e}}{1+k_{2}t}$$ (2)

Where F_t_ is $\frac{q_{t}}{q_{e}}$ , q_t_ and q_e_ are the amount of drug adsorbed at any given time, t, and at equilibrium, k is the rate constant and t is the time.

### Physicochemical characterization

The crystallographic features of the samples were investigated by X-ray diffraction (XRD) on a D8 Advance diffractometer (Bruker, Karlsruhe, Germany), with CuKα radiation, 2θ range 10–80, scan step 0.02). Fourier-transform infrared spectroscopy with attenuated total reflection (FTIR-ATR) (Nicolet iS5, Thermo Scientific) was investigated in the range 400–4,000 cm^−1^. The morphology of both scaffolds was explored by Scanning Electron Microscopy (SEM), using a Zeiss EVOMA10 scanning electron microscope (Carl Zeiss, Oberkochen, Germany) at 20 kV acceleration voltage. Inductively coupled plasma optical emission spectroscopy (ICP-OES) was used to determine the elemental composition of the samples. Elemental quantification (Ca, P and Mg) was performed by Agilent 5,100 ICP-OES spectrometer (Agilent Technologies, Santa Clara, CA, United States). Samples were prepared by dissolving 10 mg of sample powder in 50 mL of 2 wt% HNO_3_ solution. The obtained values were expressed in terms of (Ca + Mg)/P mol, Ca/P mol and Mg/Ca molar%. Standard solutions of the analysed element, obtained by dilution of certified 1,000 ppm standards (Sigma Aldrich, St. Luis, MO, United States), were used to build a concentration/emission calibration curve in the concentration range 0.1–100 ppm.

### Bacterial culture conditions

Gram-positive *Staphylococcus aureus* ATCC 25923 (*S. aureus*) bacterial strain was cultured overnight at 37 °C in 10 mL of Mueller Hinton (MH) broth (Sigma-Aldrich, United States). The number of bacterial colony-forming units (CFUs/mL) was determined by comparing the optical density (OD_600_) of the sample, with a standard curve relating the OD to bacterial CFU/mL (Restivo et al., 2024).

### Agar diffusion test

*Staphylococcus aureus* bacteria at a concentration of 10^4^ CFUs/mL, according to European Committee on Antimicrobial Susceptibility Testing (EUCAST) guidelines, were plated on MH-agar plates (Guagliano et al., 2025). To perform the agar diffusion test, HS scaffolds were cut, in sterile conditions, in round circles of 0.64 cm^2^ and functionalized with antibiotics: vancomycin 5.35 mg/scaffold, gentamicin 4.28 mg/scaffold and tobramycin 4.28 mg/scaffold. Cellulose nitrate filter discs of 0.64 cm^2^, used as control, were functionalized with the same quantity of antibiotics released from the HS scaffolds after 24 h according to the drug release curve. Moreover, the agar test was conducted on HS scaffolds and on filter discs containing PBS instead of antibiotics, which were used as negative controls. These controls were intended to demonstrate that the antibiotic-unloaded samples are not antibacterial. Furthermore, a range of drug concentrations ≥ MIC value (ranging from 2 to 200 μg/mL) were loaded onto filter discs to assess the variation of inhibition zones resulting from the different quantities of released antibiotics (data not shown). The plates were incubated for 24 h at 37 °C and the inhibition zone diameter (in mm) was measured. The experiment was performed in triplicate. Statistical analysis was conducted through a one-way analysis of variance (ANOVA), followed by Bonferroni’s test between samples.

## Results and discussion

**FIGURE 1:**
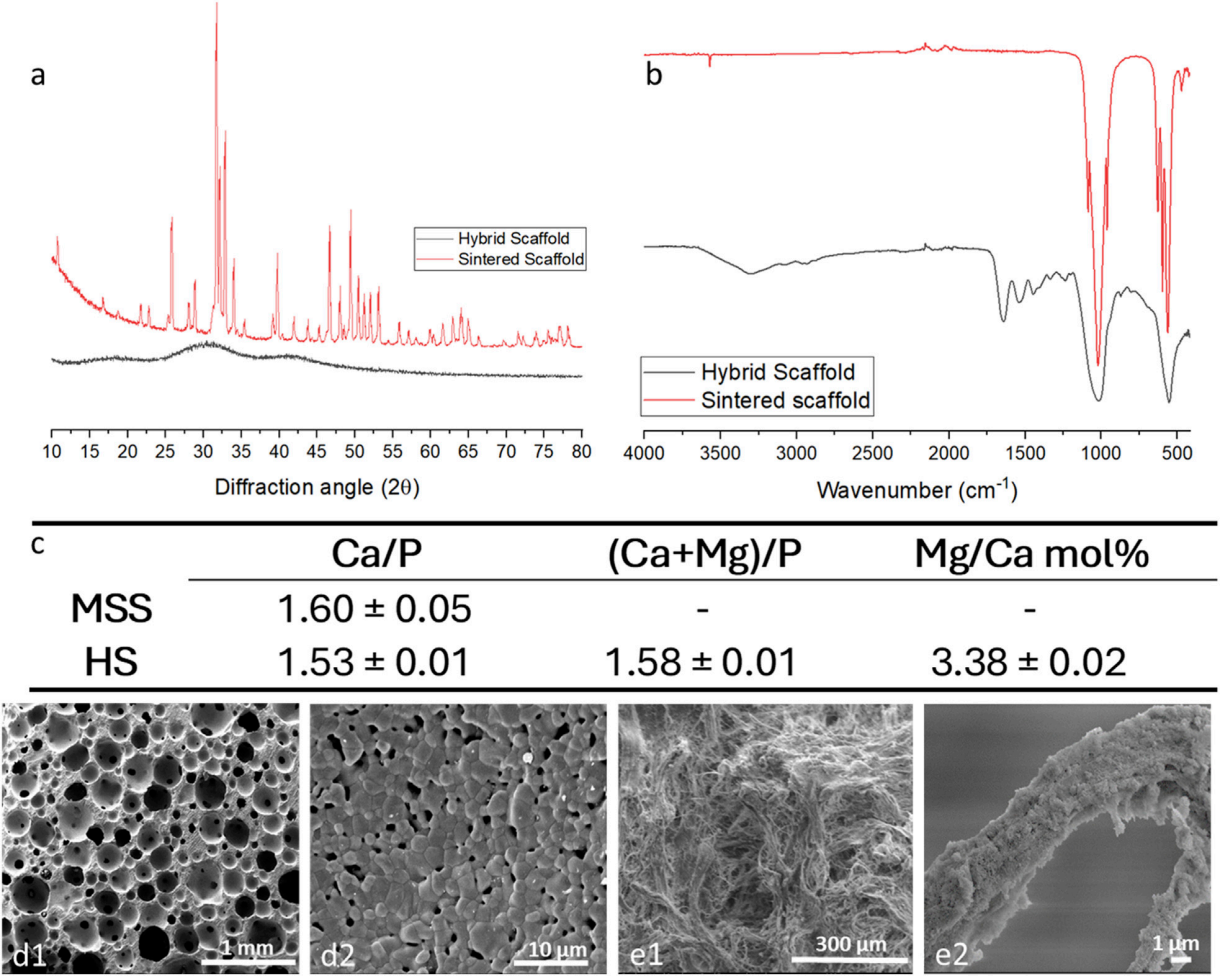
Physico-chemical and morphological analysis of MSS and HS. **(a)**: XRD pattern, **(b)** FTIR analysis, **(c)** ICP-OES analysis, d1,2) SEM micrographs of MSS and e1,2) SEM micrographs of HS.

The biomineralization process is a complex phenomenon in which natural organisms generate nanostructured hybrid inorganic/organic tissues such as bone, teeth and shells, characterized by inorganic nanocrystals grown on self-assembling bio-organic structure acting as a template guiding and controlling the heterogeneous nucleation process (Ruffini et al., 2021). In the formation of mammalian bone, collagen-based components act as a template for the heterogeneous nucleation of ion-substituted HA through chemical, physical, morphological and structural control mechanisms (Kielty and Grant, 2003; Sprio et al., 2014). Such a process was reproduced in the laboratory to obtain a hybrid scaffold with bone-mimicking composition and reproducing the woven structure of the newly formed bone tissue. Particularly, the process involved the use of Type I collagen fibrils dispersed in an aqueous solution of ions generally involved in bone formation processes, the amount of which can determine the extent of the mineral phase in the final hybrid scaffold (Sprio et al., 2012). Here the mineral phase is not simply mixed or physically embedded but is heterogeneously nucleated on the collagen matrix (Sprio et al., 2012). This implies that the formation process of mineral phase crystals is influenced by information transferred at the molecular level from the collagen matrix, resulting in physico-chemical and structural constraints which favor multiple doping with foreign ions such as Mg^2+^ and CO_3_^2-^, as well as a very reduced crystal growth, so that the mineral phase appears to be pseudo-amorphous, as shown by the broad diffraction XRD pattern (Figure 1a, black line). Conversely, the XRD analysis of MSS reported the presence of hydroxyapatite with a little amount of β-TCP (97:3 HA: β-TCP, calculated by Rietveld analysis), characterized by high crystallinity (ICDD card no. 09-0432 for HA and ICDD card no. 09-0169 for β-TCP, Figure 1a, red line) as yielded by the sintering treatment at 1,250 °C. The FTIR-ATR spectra of MSS (Figure 1b, red line) show the typical signature of crystalline HA. In particular all vibration modes of PO_4_^3-^ groups were detected, including the characteristic bands for ν_3_ and ν_1_ stretching modes and v_4_ and v_2_ bending modes at 1,087-1,032, 963, 560-600, and 472 cm^-1^, respectively, while the peaks at 632 and 3,572 cm^-1^ were attributed to the v_s_ stretching and v_L_ librational mode of the OH^−^ groups, respectively (Koutsopoulos, 2002). Concerning the HS (Figure 1b, black line), a general peak broadening of PO_4_^3-^ bands can be visible, ascribed to the presence of nearly amorphous HA phase. Furthermore, the amides (I, II, III) stretching and bending vibrations were found at 1,640, 1,545 and 1,236 cm^-1^ thus reporting to the alpha-helical structure of the collagen.

The chemical composition of both the scaffold was quantitatively evaluated by ICP-OES (Figure 1c), which indicate for both scaffold a Ca/P ratio lower than 1.67, indicating off-stoichiometry, related to the presence of foreign Mg^2+^ and CO_3_^2-^ ions partially substituting Ca^2+^ and PO_4_^3-^, or the presence of secondary phases. In particular, for the MSS scaffold the Ca/P = 1.60 ± 0.05 can be related to the presence of 3% βTCP. On the other hand, in the case of HS, the Ca/P = 1.53 ± 0.01 and (Ca + Mg)/P = 1.58 ± 0.01 ratios, both lower than the theoretical one, indicate that the Mg, CO_3_ co-doped HA mineral phase is calcium-deficient and that Mg ions partially substituted Ca^2+^ in the HA structure. In this respect, the Mg^2+^/Ca^2+^ molar ratio in the mineral phase was found equal to 3.38 mol%, indicating that the presence of magnesium ions is at a level comparable to that of biogenic HA (natural bone).

The morphology of both scaffolds was evaluated by SEM, as shown in Figure 1d1,2 and e1,2. The SEM analysis of MSS (Figure 1d1) shows the coalescence of the HA grains attesting structural consolidation, with a high extent of interconnected spherical macro-pores, interspersed with micrometric intergranular pores (Figure 1d2). The morphology of the HS (Figure 1e1,e2) shows a highly heterogeneous porous and fibrous arrangement with relatively large and small agglomerates of hydroxyapatite nanocrystals tightly bound to collagen fibres.

As previously reported, the mean porosity of the MSS, as determined by the Archimedes’ method, was 83.1% ± 0.2%, a finding that was corroborated by mercury porosimetry analysis giving a porosity extent of 84%, while revealing a bimodal distribution of pores with an average diameter of 218 ± 8 µm and a modal diameter of 742 ± 2 µm (Dapporto et al., 2016). In contrast, the overall porosity of HS was about 82.4% ± 0.3% with anisotropic pore size ranging from 50 to 200 µm (Krishnakumar et al., 2018). The compressive strength of both MSS and HS scaffold was previously measured to be 1.7 ± 0.4 MPa and 13.9 ± 2.8 KPa, respectively (Dapporto et al., 2016; Krishnakumar et al., 2018).

**FIGURE 2:**
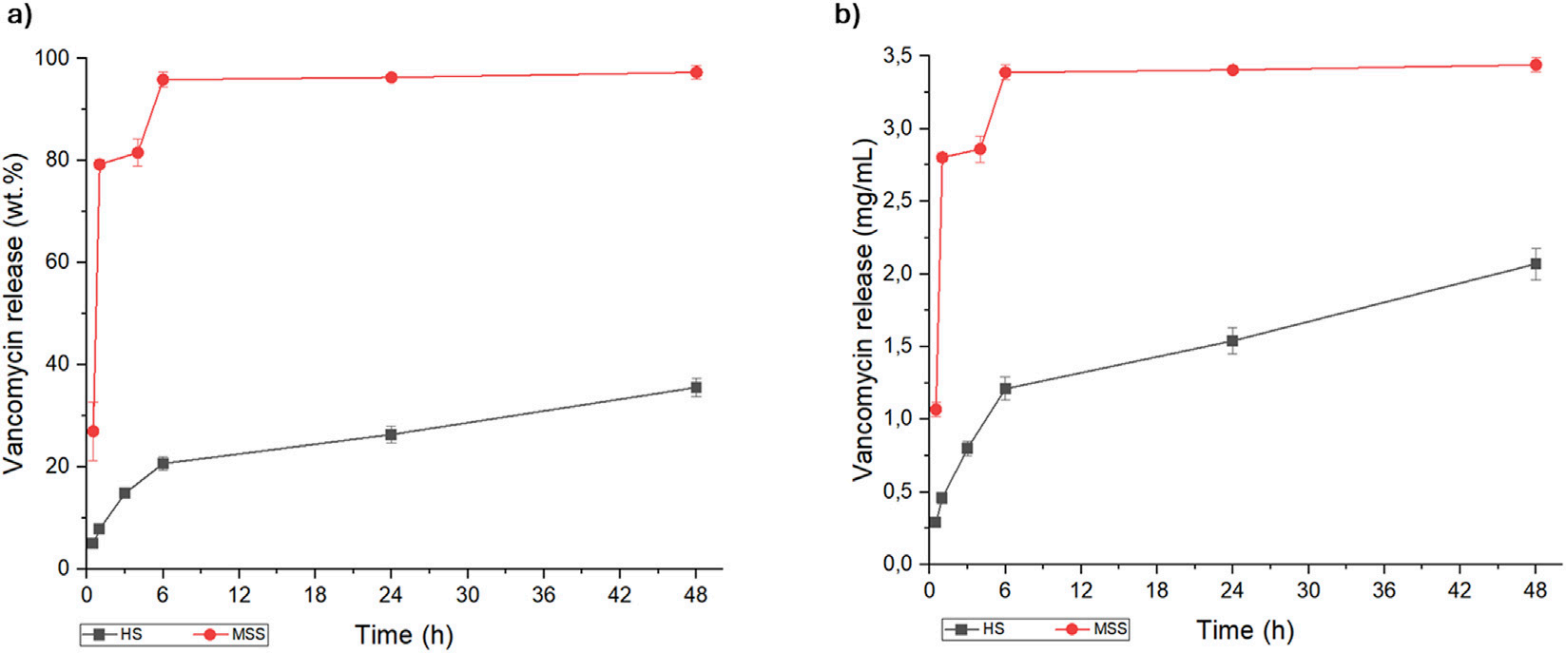
Kinetic release of vancomycin from macroporous sintered scaffold (red line) and hybrid scaffold (black line), expressed as **(a)** weight percentage (wt.%) and **(b)** concentration (mg/mL).

The present study investigates the efficacy of HS as an efficient drug delivery system for the sustained release of antibiotics. VNC, GNT or TBR were successfully loaded onto the HS surface through fast and simple procedure, enabling the immediate preparation of medicated implants in the operatory theatre prior to surgical intervention. The release profile of VNC from the HS was initially evaluated in comparison with that from a MSS, with the aim of assessing the influence of the microstructure and the physicochemical features of the scaffolds under physiological conditions. In particular, the VNC loading on both the scaffolds was performed by dropwise settling of 50 mg/mL of drug solution onto the scaffold. The release test of VNC from MSS and HS was performed for up to 48 h in PBS medium (pH 7.4) at 37 °C, under constant oscillation (Figure 2). This timelapse was chosen as an incubation time of 48 h is widely considered as sufficient and acceptable for evaluating the antibacterial activity of a drug *in vitro* (Egervärn et al., 2007).

The minimum inhibitory concentration (MIC) of VNC against MRSA is about 0.78–3.12 mg/L (Watanakunakorn and Tisone, 1982; Mulazzi et al., 2021), therefore it is important that the initial local release is above the MIC, in order to ensure the eradication of the present bacteria and the prevention of bacteria adhesion on the scaffold surface. For both scaffolds, the amount of VNC released was higher than MIC; on the other hand, the VNC release kinetic profiles were significantly different in the MSS and HS scaffolds. These differences can be ascribed to different factors, priory related to the physico-chemical properties of the scaffold determining its interaction with the antibiotic. The scaffold composition and structure, in terms of porosity extent, pore size distribution and interconnectivity, are the most important features that may have a major impact on the drug uptake and the release (Parent et al., 2017; Uskoković, 2019; Fosca et al., 2022). In this respect, both MSS and HS are based on hydroxyapatite, however the different synthesis method yielded 3D scaffolds featuring substantial differences in terms of chemical composition, crystalline features, nanostructure and porosity. In spite the MSS shows a wide interconnected macro-porosity and a relatively high specific surface area (as shown in Figure 1d1), its ability to retain the drug is comparatively diminished with respect to the HS. This is attributable to the consequences of the thermal sintering process, which results in an enhancement of crystallinity and of grain growth related to a reduced ability to effectively link the drugs. We can hypothesize that the interaction of the antibiotics with MSS is based on weak Van der Waals forces, resulting in a burst release (De Carvalho et al., 2019) and explaining the complete delivery of VNC within 1 h. In contrast, the HS, which is the result of a biomineralization process carried out at body temperature, has several charged groups exposed by the collagen and by the apatite nanocrystals, permitting a stronger interaction with VNC. Such links, together with multimodal and interconnected porosity, allow a drug release process sustained over time and characterized by a slower kinetics.

**FIGURE 3:**
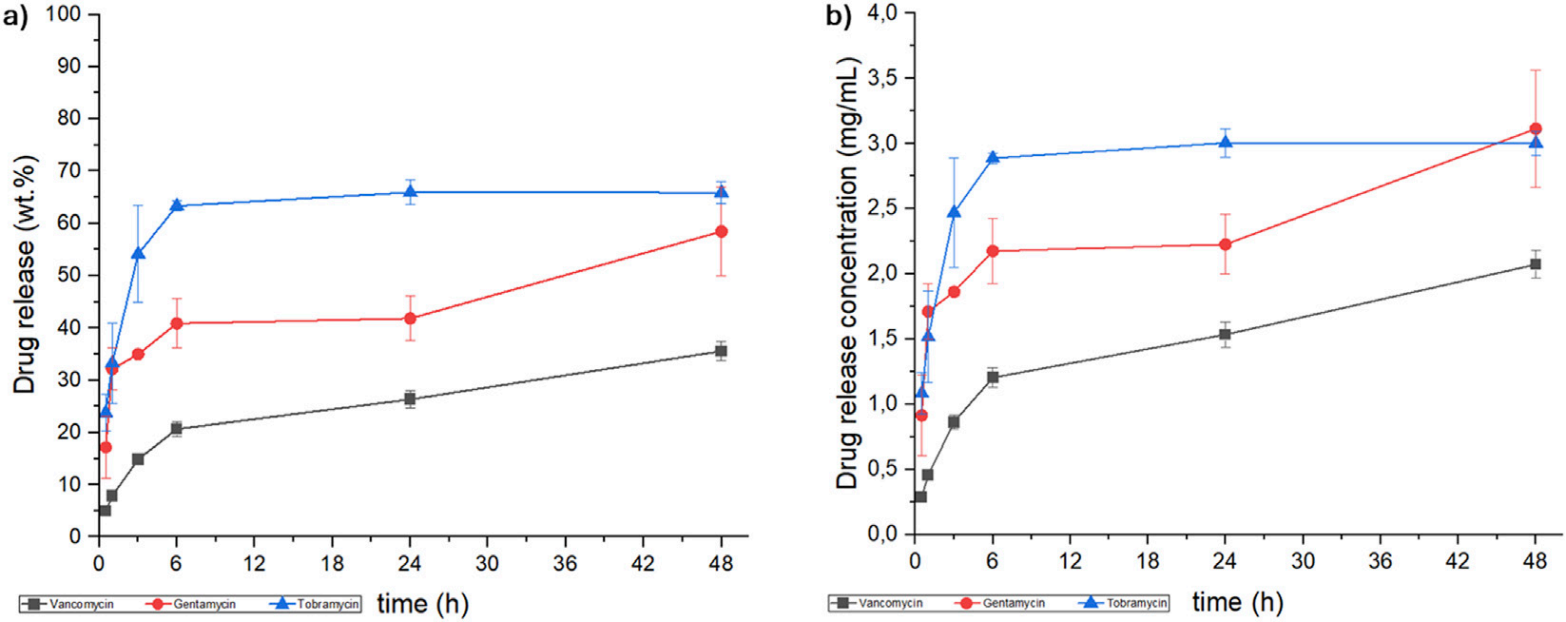
Kinetic release of vancomycin, (black line), gentamicin (red line) and tobramycin (blue line) from hybrid scaffold, expressed as **(a)** weight percentage (wt.%) and **(b)** concentration (mg/mL).

Based on these first results, the evaluation of the release of two aminoglycosidic antibiotics (GNT and TBR) was assessed exclusively using the HS scaffold, up to 48 h in PBS medium (pH 7.4) at 37 °C, under constant oscillation. The GNT and TBR release profiles, compared to that of VNC, are shown in Figure 3.

As demonstrated in Figure 3, the concentration of all the antibiotics released reached the MIC for the MRSA strain (VNC: 0.78–3.12 mg/L (Watanakunakorn and Tisone, 1982; Mulazzi et al., 2021), GNT: 1.56 - >25 mg/L (Watanakunakorn and Tisone, 1982) and TRB: 6.25 - >25 mg/L (Watanakunakorn and Tisone, 1982)). Despite identical scaffold composition and processing conditions, the release kinetics exhibited significant differences, which were further clarified by fitting the release data using pseudo-first and pseudo-second order kinetic models (Equation 1; Equation 2). These variations can be ascribed to the molecular interaction between antibiotics and the collagen/hydroxyapatite surface of HS, in particular related to the different hydrophobicity/hydrophilicity, molecular weight and steric bulk of the antibiotics (Li and Mooney, 2016; Parent et al., 2017; Uskoković, 2019; Fosca et al., 2022). In this regard, the release profiles showed that VNC and GNT followed pseudo-second order kinetics, suggesting scaffold-drug interactions are the rate-determining step. Conversely, TBR followed pseudo-first order kinetics, suggesting that its release is primarily dependent on the drug concentration into the scaffold and controlled by diffusion (Bruschi, 2015; Paarakh et al., 2019; Hong et al., 2024).

**FIGURE 4:**
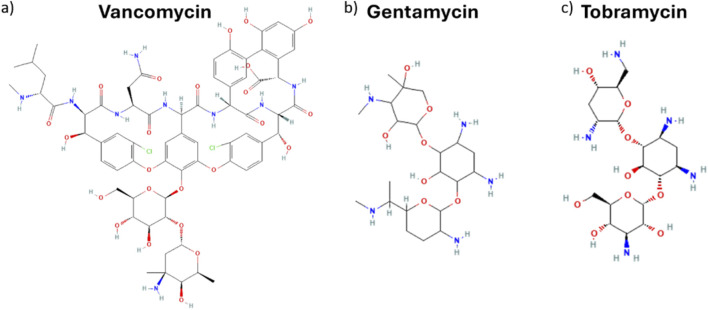
Chemical structures of **(a)** Vancomycin, **(b)** Gentamicin, and **(c)** Tobramycin.

Fitting-derived kinetic and equilibrium parameters shed further light on critical factors influencing drug release behaviour. A slower and less consistent release was reported for VNC compared to GNT, both fitting with the same kinetic order. Such tendency is reflected in both lower rate constant k_2_ and equilibrium drug release ratio q_e_ (Table 1) for VNC with respect to GNT. The reduced values can be ascribed to VNC’s distinct molecular feature that, from a chemical point of view, is a glycopeptide antibiotic (molecular weight of about 1.45 KDa), characterized by multiple functional groups, including phenolic, carboxyl and amine moieties (Figure 4a). On the other hand, GNT and TBR are aminoglycosidic antibiotics (molecular weight of about 477 and 467 Da respectively) (Figures 4b,c respectively) exhibiting similar chemical structures, characterized by a 2-deoxystreptamine core linked to multiple amino sugars. In both VNC and GNT, hydroxyl and amino groups can participate in hydrogen bonding with functional groups within the collagen/hydroxyapatite matrix (Singh et al., 1995). Moreover, under physiological conditions, functional groups on both VNC and GNT can undergo protonation, resulting in a net positive charge, which leads to the establishment of isotropic electrostatic interaction with negatively charged carboxyl groups on the collagen matrix or with phosphate groups in hydroxyapatite nanocrystals (Singh et al., 1995; Jia et al., 2013; Kilb et al., 2022). As a result, the slower VNC release compared to GNT can be attributed to its greater number of functional groups capable of forming hydrogen bonds and electrostatic interactions with the HS scaffold. Although both antibiotics can engage in multiple interactions under physiological conditions, VNC’s structural complexity favours more extensive binding, thereby reducing its release rate into the aqueous medium.

**TABLE 1:** Kinetic order applied to the antibiotics release, calculated constant (k) and equilibrium drug release ratio (q_e_) and R^2^.

Antibiotics	Kinetic order	Calculated k	Calculated q_e_	*R* ^*2*^
Vancomycin	Pseudo-second order kinetic	k_2_ = 0.31 ± 0.05	0.33 ± 0.03	0.98
Gentamicin	Pseudo-second order kinetic	k_2_ = 1.73 ± 0.70	0.45 ± 0.04	0.79
Tobramycin	Pseudo-first order kinetic	k_1_ = 0.82 ± 0.10	0.62 ± 0.01	0.99

On the other hand, TBR seems to follow a different release mechanism to the one observed for GNT. Although they have a similar structure, TBR and GNT present some subtle but significant differences in terms of physicochemical properties. Actually, differences in aqueous solubility have been demonstrated to exert a significant influence on the process of release. Drugs exhibiting lower solubility demonstrate a slower release rate due to their higher affinity with the hydrophobic domain of the collagen matrix (Li and Mooney, 2016; Parent et al., 2017; Uskoković, 2019; Fosca et al., 2022). The partition coefficient, log P, is a crucial parameter in predicting the pharmacokinetic behaviour, facilitating the interpretation of the observed release for the studied antibiotics (Xing and Glen, 2002; Verma et al., 2011). However, it is necessary to acknowledge that the log P value is related to the neutral state of molecules, while it becomes pH dependent when the presence of basic or acidic functional groups in drugs is observed. The pH-dependent distribution coefficient, log D, provides a more precise representation of drug behaviour in various biological environments. In a manner analogous to log P, the higher the value of log D, the more lipophilic the compound. Conversely, lower values indicate higher hydrophilicity (and consequently greater water solubility) (Xing and Glen, 2002). Although both GNT and TBR present high solubility in water, at physiological pH, the log D of VNC, GNT and TBR are −5.14 (ChemSpider, 2025c), −7.90 (ChemSpider, 2025a) and −9.45 (ChemSpider, 2025b) respectively. Consequently, TBR demonstrates a higher tendency to diffuse into the aqueous phase than GNT and VNC. While the release process of GNT and VNC is largely controlled by their interactions with the scaffold matrix, as evidenced by pseudo-second-order kinetics that show chemisorption-limited processes, the release of TBR is primarily controlled by diffusion into the surrounding medium, according to a pseudo-first-order model. Such a result could imply weaker interactions of TBR with the scaffold and its higher mobility in solution. The high equilibrium release capacity (q_e_) seen in TBR with respect to GNT and VNC corroborates this rationale, aligning with a faster release attributed to its enhanced aqueous compatibility and decreased scaffold interaction.

These findings indicate that while log D provides essential insights about release potential, it should not be evaluated alone. A comprehensive understanding of drug release behaviour requires the integration of several characteristics, including steric properties, molecular conformation, solubility, and specific interactions with the scaffold matrix.

This latter aspect assumes a specific relevance when the scaffold is endowed with a chemically active surface as occurs with non-sintered and structurally disordered phases such as the hybrid scaffold of the present study. An interesting aspect in this regard is that features strictly associated to the scaffold bioactivity such as nanocrystallinity, nanostructure and high porosity, are likewise related to the capacity of the scaffold to retain and release therapeutics along sustained kinetic profiles. This suggests that the use of active scaffolds obtained by bio-inspired processes may be advantageous to obtain devices with multifunctional ability relevant to contrast infections along with effective regenerative processes.

**FIGURE 5:**
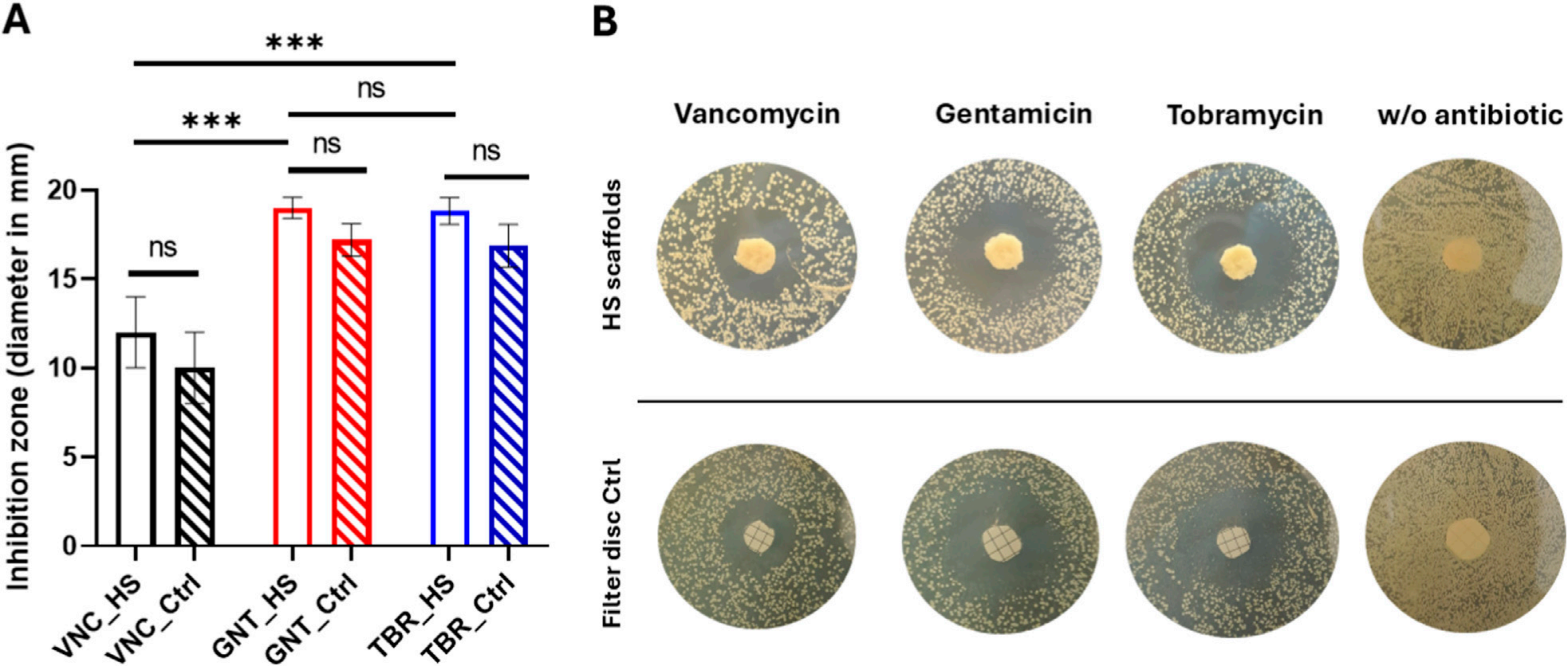
Antibacterial effect of antibiotics released from HS scaffolds after 24 h in comparison with the filter disc control. Inhibition zone diameter **(A)** and agar plates **(B)**. No bacterial inhibition was observed on HS scaffolds/filter discs without antibiotics **(B)**.

Microbiological tests were carried out by analysing the three different antibiotic drugs released by the HS scaffolds with Gram-positive *Staphylococcus aureus* through the agar diffusion test. As illustrated in Figure 5, the inhibition zone diameter (in mm) is demonstrated after 24 h of drug release (panel A). Panel B of the figure showed the inhibition zones on the agar plates. Data in Figure 5A were represented as the mean of triplicates ±standard deviation. The results indicate that all the antibiotics released from the scaffolds after 24 h exhibited antibacterial activity, although the aminoglycosidic antibiotics gentamicin and tobramycin demonstrated a superior efficacy compared to vancomycin (p < 0.05). The lower inhibition zone of VNC (approximately 12 mm) in comparison to GNT and TBR (approximately 19 mm) can be ascribed not solely to the low release kinetics of this drug (see Figure 3) but also to the reduced susceptibility of *S. aureus* bacteria to VNC (Ajit Singh et al., 2019) in comparison to the other antibiotics. This reduced susceptibility is attributable to the distinct permeability of VNC into bacterial cells. No statistically significant differences between GNT and TBR were observed (p > 0.05). Furthermore, the same quantity of drugs released from the HS scaffolds after 24 h (as illustrated in Figure 3), was loaded on filter discs, used as controls, to ascertain the antibacterial effect of the released antibiotics. As demonstrated in Figure 5A no statistically significant differences (p > 0.05) in antibacterial properties were observed between the various HS scaffolds and their disc controls loaded with antibiotics. In addition, negative controls represented by either HS scaffolds or filter disc without antibiotics (Figure 5B) were added to demonstrate that the antibiotic-unloaded HS scaffold/filter disc do not have an antibacterial effect.

Our results demonstrate the effectiveness of antibiotic-loaded hybrid bone scaffolds prepared with simple procedures suitable to be performed in the operatory arena. This is significant, in the perspective of reducing the systemic administration of antibiotics to combat bone infections, whereas *in situ* delivery will permit to enhance the local dose of antibiotic and its efficacy, whilst reducing at the same time adverse side effects related to drug toxicity and, most importantly, to contrast the bacterial resistance to antibiotics, which is among the major cause of surgical therapies failure.

## Conclusion

The continuous increase of orthopaedic infections, associated with ever rising bacterial resistance to antibiotics, is pushing the research on bone scaffolds able to exert both regenerative ability and drug delivery function. In this respect, the present work highlights the suitability of bioactive hybrid scaffolds featuring biomimetic composition, crystallinity, nanostructure and porosity, as a delivery system able to release various clinically relevant antibiotics: vancomycin, gentamicin, and tobramycin, thus promising to promote bone tissue regeneration whereas contrasting infections. The chemically active surface and nanostructure exhibited by the scaffold allowed efficient loading of the drug and modulated release kinetics, which were influenced by the specific molecular characteristics of the antibiotic. These results indicate the potential for hybrid scaffolds, and more generally for bio-devices exhibiting chemically active surface and multi-scale porosity, to function as an effective multifunctional platform for promoting bone regeneration and efficient on-site antibiotic-based therapies.

Considering that the occurrence of nosocomial infections is increasingly rising, future investigations should extend the antimicrobial testing to encompass a wider range of bacterial strains involved with in vivo infections. In this respect, since complex clinical cases such as bone cancer or osteoporosis may require the administration of multiple therapeutic agents to support bone regeneration, it will be important to explore the local co-administration of multiple therapeutic agents by bone scaffolds. This is certainly a great challenge for material scientists and developers, as *in situ* delivery will have to be made with quite accurate drug-dependent time-resolved profiles. However, following this pathway may bring to results of invaluable importance in medicine, enabling more appropriate and rationale dosage of drugs greatly reducing adverse effects.

## Data Availability

The raw data supporting the conclusions of this article will be made available by the authors, without undue reservation.
